# Multimodal machine learning for predicting postoperative functional outcomes in surgically treated supratentorial deep intracerebral hemorrhage: a prospective multicenter study

**DOI:** 10.3389/fneur.2026.1774621

**Published:** 2026-03-05

**Authors:** Min Cui, Yanyi Liu, Qi He, Weiming Xiong, Yang Liu, Lei Xu, Yongbing Deng, Xingwei Tan

**Affiliations:** 1 Department of Neurosurgery, Chongqing Emergency Medical Center, Chongqing University Central Hospital, Chongqing, China; 2 Department of Neurosurgery, Yongchuan Hospital of Chongqing Medical University, Chongqing, China; 3 Department of Neurological Disease Center, The Third Affiliated Hospital of Chongqing Medical University(FangDa Hospital), Chongqing, China

**Keywords:** biomarkers, intracerebral hemorrhage, machine learning, modified rankin scale, random forest, SHAP, supratentorial deep hemorrhage

## Abstract

**Background:**

Early prediction of functional outcomes after surgery for spontaneous supratentorial deep intracerebral hemorrhage (sICH) remains difficult. This study developed and validated multimodal machine-learning models incorporating clinical, imaging, physiological, and biomarker data, including temperature management strategies, and explored interpretability using SHAP.

**Methods:**

This prospective multicenter cohort enrolled 285 surgically treated sICH patients. Outcome was defined as favorable (mRS 0–3) vs. unfavorable (mRS 4–6). Data were split by stratified random sampling into a training set (*n* = 199) and a test set (*n* = 86). LASSO with 10-fold cross-validation (1-SE rule) selected key predictors. Five classifiers (Random Forest, neural network, decision tree, k-nearest neighbors, naïve Bayes) were trained with 10-fold cross-validation and evaluated on the test set. Performance was assessed using AUC (95% CI) and standard classification metrics; AUCs were compared by DeLong's test. SHAP was applied to the best model.

**Results:**

LASSO identified eight predictors: admission GCS, hematoma volume, TNF-α, GFAP, IL-1β, admission NIHSS, mean body temperature, and peak ICP. On the test set, Random Forest achieved the highest performance (AUC 0.883, 95% CI 0.829–0.937; accuracy 0.824; F1-score 0.836), with no significant AUC difference versus the neural network (AUC 0.867; *P* = 0.312). SHAP ranked admission GCS and hematoma volume as the most important features, followed by TNF-α and GFAP.

**Conclusions:**

A multimodal Random Forest model provided good discrimination for predicting postoperative functional outcomes in surgically treated sICH, and SHAP improved interpretability by quantifying feature contributions.

## Introduction

Spontaneous intracerebral hemorrhage (ICH) is one of the most disabling and fatal forms of acute cerebrovascular disease in adults (1). Although it accounts for approximately 10–15% of all stroke events, the associated mortality and long-term disability rates remain as high as 40–50% (2). Among these, supratentorial deep intracerebral hemorrhage (sICH) is associated with particularly poor outcomes and is frequently accompanied by rapid neurological deterioration, elevated intracranial pressure, and systemic complications, posing substantial challenges for clinical management and prognostic assessment (3, 4). In recent years, postoperative normothermia control has been shown to have potential in improving the prognosis of patients with traumatic brain injury and spontaneous subarachnoid hemorrhage. Although its efficacy in sICH patients is not yet well-established, normothermia control has been recommended in some consensus guidelines for the clinical management of spontaneous intracerebral hemorrhage, suggesting it may help reduce neurological damage, protect neurological function, and improve postoperative recovery. Proper temperature management can effectively control temperature fluctuations, reduce secondary brain injury, and thus improve the patient's quality of life and recovery speed (4).

Currently, prognostic evaluation in routine practice mainly relies on conventional clinical grading systems, such as the Glasgow Coma Scale (GCS) and the National Institutes of Health Stroke Scale (NIHSS), as well as imaging characteristics including hematoma volume, intraventricular hemorrhage (IVH), and midline shift. However, when used in isolation, these indicators often fail to fully capture the complexity of disease progression and inter-individual heterogeneity, resulting in limited predictive accuracy. In addition, inflammatory and neuronal injury biomarkers (e.g., TNF-α, IL-1β, GFAP, S100β, and NSE) play important roles in the pathophysiology of secondary brain injury after ICH, reflecting inflammatory responses and the extent of tissue damage, and may therefore provide added value for preoperative risk stratification (5, 6).

In recent years, machine learning (ML) has demonstrated considerable potential in medical prognostic modeling by integrating multidimensional data and capturing complex non-linear relationships among variables, offering new opportunities for individualized outcome prediction (7). Prior studies have shown that algorithms such as random forests and neural networks can achieve favorable performance in predicting outcomes after stroke and ICH, improving discrimination while maintaining relatively good model stability (8, 9). Nevertheless, existing work has often focused on single-modality data or single-center cohorts, and interpretable, multimodal, multicenter-integrated models remain scarce, limiting direct clinical translation.

The study evaluates the effect of postoperative normothermia control on the improvement of the 3-month mRS score and constructs a predictive model based on inflammation and neuroinjury biomarkers. The best model is selected for prognosis prediction, providing support for clinical treatment decisions. By integrating clinical variables, imaging features, and biomarkers, the study aims to develop a machine learning-based prognostic model, using SHAP to enhance model interpretability and provide evidence for preoperative risk stratification and personalized treatment decisions.

## Method

### Study design and participants

This was a prospective, multicenter cohort study. Patients with spontaneous supratentorial deep intracerebral hemorrhage (sICH) who presented to and underwent surgical treatment at Chongqing University Central Hospital were consecutively enrolled. All patients completed clinical data collection, neuroimaging assessment, serum biomarker testing, and were managed according to temperature control protocols. They were included in the final analysis (*n* = 285).

### Eligibility criteria

**Inclusion criteria** were: (1) CT-confirmed spontaneous supratentorial deep parenchymal hemorrhage (basal ganglia or thalamus); (2) receipt of surgical intervention (craniotomy for hematoma evacuation or minimally invasive surgery); (3) completion of blood sampling and baseline examinations within 24 h of admission; and (4) availability of complete follow-up outcome data.

**Exclusion criteria** were: (1) secondary intracerebral hemorrhage (e.g., arteriovenous malformation, tumor, aneurysmal rupture, trauma); (2) severe infection, autoimmune disease, or long-term immunosuppressive therapy that could affect inflammatory indices; (3) severe hepatic or renal failure, or malignancy; and (4) substantial missing data or loss to follow-up.

### Clinical and perioperative data collection

Demographic characteristics and medical history at admission (age, sex, hypertension, diabetes mellitus, smoking, alcohol consumption), vital signs (systolic/diastolic blood pressure), neurological severity scores [Glasgow Coma Scale (GCS) and National Institutes of Health Stroke Scale (NIHSS)], and perioperative variables (time from onset to surgery and surgical approach: craniotomy vs. minimally invasive surgery) were collected. Perioperative physiological parameters were also recorded, including mean body temperature, peak intracranial pressure (ICP), mean cerebral perfusion pressure (CPP), and transcranial Doppler (TCD) parameters (e.g., mean flow velocity and pulsatility index).

### Neuroimaging assessment

All patients underwent head computed tomography (CT) after admission. Imaging assessments were independently performed by two qualified neuroradiologists who were blinded to clinical data and outcomes. In cases of disagreement, a third senior neuroradiologist reviewed the images and made the final decision, and consensus results were used for analysis. Hematoma volume was quantitatively measured on admission CT using the standardized ABC/2 method: A represents the greatest hemorrhage diameter on the slice with the largest area, B the diameter perpendicular to A on the same slice, and C the number of slices containing hemorrhage multiplied by slice thickness (or estimated thickness); hematoma volume ≈ A × B × C/2 (ml). Intraventricular hemorrhage (IVH) was recorded as present/absent. Midline shift was measured on standard axial CT as the displacement of midline structures (mm) and dichotomized using a threshold of >5 mm. Hemorrhage location was classified as basal ganglia or thalamus based on CT.

### Inflammatory and neuronal injury biomarker measurements

Peripheral venous blood was collected within 24 h of admission. Serum was separated by centrifugation, aliquoted, and stored at −80 °C, avoiding repeated freeze–thaw cycles. Batch testing was performed to reduce inter-assay variation. Inflammatory biomarkers TNF-α and IL-1β were quantified using commercial enzyme-linked immunosorbent assay (ELISA) kits, following the manufacturers' instructions and standard curve procedures. The neutrophil-to-lymphocyte ratio (NLR) was calculated from the admission complete blood count as neutrophil absolute count divided by lymphocyte absolute count. Neuronal injury biomarkers GFAP, S100β, and NSE were also quantified using commercial ELISA kits.

Quality control measures included the use of low- and high-concentration quality controls and repeated testing of a subset of samples to assess repeatability. Laboratory personnel were blinded to clinical outcomes during testing and data entry.

### Outcome definition and follow-up

The primary outcome was the modified Rankin Scale (mRS) score at follow-up. According to the prespecified dichotomization, mRS 0–3 was defined as a favorable outcome and mRS 4–6 as an unfavorable outcome. Follow-up was conducted in a standardized manner by trained personnel, and data were checked for completeness and consistency before analysis.

### Statistical analysis and machine learning procedures

All analyses were performed using **R software** (R Foundation for Statistical Computing, Vienna, Austria). Continuous variables were tested for normality. Normally distributed variables are presented as mean ± standard deviation and compared using the independent-samples *t* test; non-normally distributed variables are presented as median (interquartile range) and compared using the Mann–Whitney U test. Categorical variables are presented as number (percentage) and compared using the chi-square test or Fisher's exact test, as appropriate. All tests were two-sided, and *P* < 0.05 was considered statistically significant.

For machine learning, the dataset was split into a training set (*n* = 199) and an independent test set (*n* = 86) using stratified random sampling to preserve outcome proportions. In the training set, feature selection was performed using least absolute shrinkage and selection operator (LASSO) regression with 10-fold cross-validation, and the optimal penalty parameter was determined using the 1-standard-error (1-SE) rule. Five classification models were then developed and compared, including Random Forest, Neural Network, Decision Tree, K-Nearest Neighbors, and Naïve Bayes; hyperparameters were optimized using 10-fold cross-validation. Model performance was evaluated on the independent test set by reporting AUC (95% confidence interval), accuracy, sensitivity, specificity, F1-score, positive predictive value (PPV), and negative predictive value (NPV). AUCs were compared using DeLong's test, and receiver operating characteristic (ROC) curves and calibration curves were plotted. For interpretability, SHapley Additive exPlanations (SHAP) was applied to the best-performing.

## Results

### 3.1 Study cohort and data partitioning

A total of 285 patients with spontaneous supratentorial deep intracerebral hemorrhage (sICH) who underwent surgical intervention were enrolled in this prospective multicenter cohort study. The dataset was randomly divided into a training set (*n* = 199, 69.8%) and a test set (*n* = 86, 30.2%) using stratified random sampling to maintain consistent outcome distribution between sets. In the training set, 108 patients (54.3%) achieved favorable outcomes (mRS 0–3), while 91 (45.7%) had unfavorable outcomes (mRS 4–6). The test set showed similar distribution with 47 (54.7%) favorable and 39 (45.3%) unfavorable outcomes.

### 3.2 Baseline characteristics and biomarker comparison

Table 1 presents the comprehensive baseline characteristics, inflammatory markers, and neuronal injury biomarkers comparing patients with favorable vs. unfavorable outcomes in the entire cohort. Patients with unfavorable outcomes were significantly older (mean age 64.3 ± 11.2 vs. 58.7 ± 10.5 years, *P* < 0.001), had lower admission Glasgow Coma Scale (GCS) scores [median 8 (IQR 6–10) vs. 11 (IQR 9–13), *P* < 0.001], and higher National Institutes of Health Stroke Scale (NIHSS) scores [median 18 (IQR 15–22) vs. 12 (IQR 9–15), *P* < 0.001].

**Table 1:** Baseline characteristics, inflammatory markers, and neuronal injury biomarkers.

**Variable**	**Favorable outcome (mRS 0-3) *n* = 155**	**Unfavorable outcome (mRS 4–6) *n* = 130**	***P* value**
**Demographics**			
Age (years)	58.7 ± 10.5	64.3 ± 11.2	< 0.001
Male sex	96 (61.9%)	77 (59.2%)	0.642
Hypertension	113 (72.9%)	105 (80.8%)	0.128
Diabetes mellitus	43 (27.7%)	43 (33.1%)	0.336
Current smoking	59 (38.1%)	55 (42.3%)	0.474
Alcohol consumption	52 (33.5%)	48 (36.9%)	0.558
**Clinical presentation**			
Admission GCS score	11 (9–13)	8 (6–10)	< 0.001
Admission NIHSS score	12 (9–15)	18 (15–22)	< 0.001
Systolic BP (mmHg)	168 ± 24	175 ± 28	0.024
Diastolic BP (mmHg)	94 ± 16	98 ± 18	0.062
**Imaging characteristics**			
Hematoma volume (**ml**)	32.6 ± 14.7	45.8 ± 18.3	< 0.001
Intraventricular hemorrhage	65 (41.9%)	89 (68.5%)	< 0.001
Midline shift >5 mm	80 (51.6%)	97 (74.6%)	< 0.001
Basal ganglia location	96 (61.9%)	78 (60.0%)	0.738
Thalamic location	59 (38.1%)	52 (40.0%)	0.738
**Surgical factors**			
Time to surgery (hours)	8.5 (6.0–12.0)	9.0 (6.5–13.5)	0.187
Craniotomy	116 (74.8%)	103 (79.2%)	0.390
Minimally invasive surgery	39 (25.2%)	27 (20.8%)	0.390
**Inflammatory markers**			
TNF-α (pg/**ml**)	28.4 (22.1–36.7)	45.6 (35.2–58.3)	< 0.001
IL-1β (pg/**ml**)	22.5 (16.8–30.2)	38.9 (28.7–52.1)	< 0.001
NLR	5.2 (3.8–7.1)	8.9 (6.2–12.4)	< 0.001
**Neuronal injury markers**			
GFAP (ng/**ml**)	9.3 (6.7–13.5)	15.8 (11.2–22.4)	< 0.001
S100β (μg/L)	0.52 (0.38–0.71)	0.85 (0.62–1.18)	< 0.001
NSE (ng/**ml**)	17.2 (12.8–23.5)	28.6 (21.4–38.7)	< 0.001
**Physiological parameters**			
Mean body temperature (°C)	37.2 ± 0.4	37.8 ± 0.6	< 0.001
Peak ICP (mmHg)	18.5 ± 6.3	26.8 ± 8.7	< 0.001
Mean CPP (mmHg)	72.4 ± 8.9	65.3 ± 10.2	< 0.001
TCD mean flow velocity (cm/s)	58.3 ± 14.2	48.7 ± 16.8	< 0.001
TCD pulsatility index	0.92 ± 0.18	1.15 ± 0.24	< 0.001

Hematoma volume was substantially larger in the unfavorable outcome group (45.8 ± 18.3 ml vs. 32.6 ± 14.7 ml, *P* < 0.001), with more frequent intraventricular hemorrhage (IVH) extension (68.6% vs. 42.1%, *P* < 0.001) and midline shift >5 mm (74.8% vs. 51.6%, *P* < 0.001). Inflammatory markers and neuronal injury biomarkers all demonstrated significant differences between groups. Tumor necrosis factor-alpha (TNF-α) levels were markedly elevated in patients with unfavorable outcomes (median 45.6 [IQR 35.2–58.3] vs. 28.4 [IQR 22.1–36.7] pg/ml, *P* < 0.001), as were interleukin-1 beta (IL-1β) [median 38.9 (IQR 28.7–52.1) vs. 22.5 (IQR 16.8–30.2) pg/ml, *P* < 0.001], neutrophil-to-lymphocyte ratio (NLR) [median 8.9 (IQR 6.2–12.4) vs. 5.2 (IQR 3.8–7.1), *P* < 0.001], glial fibrillary acidic protein (GFAP) [median 15.8 (IQR 11.2–22.4) vs. 9.3 (IQR 6.7–13.5) ng/ml, *P* < 0.001], S100β [median 0.85 (IQR 0.62–1.18) vs. 0.52 (IQR 0.38–0.71) μg/L, *P* < 0.001], and neuron-specific enolase (NSE) [median 28.6 (IQR 21.4–38.7) vs. 17.2 (IQR 12.8–23.5) ng/ml, *P* < 0.001].

### 3.3 Feature selection and variable importance

**Figure 1:**
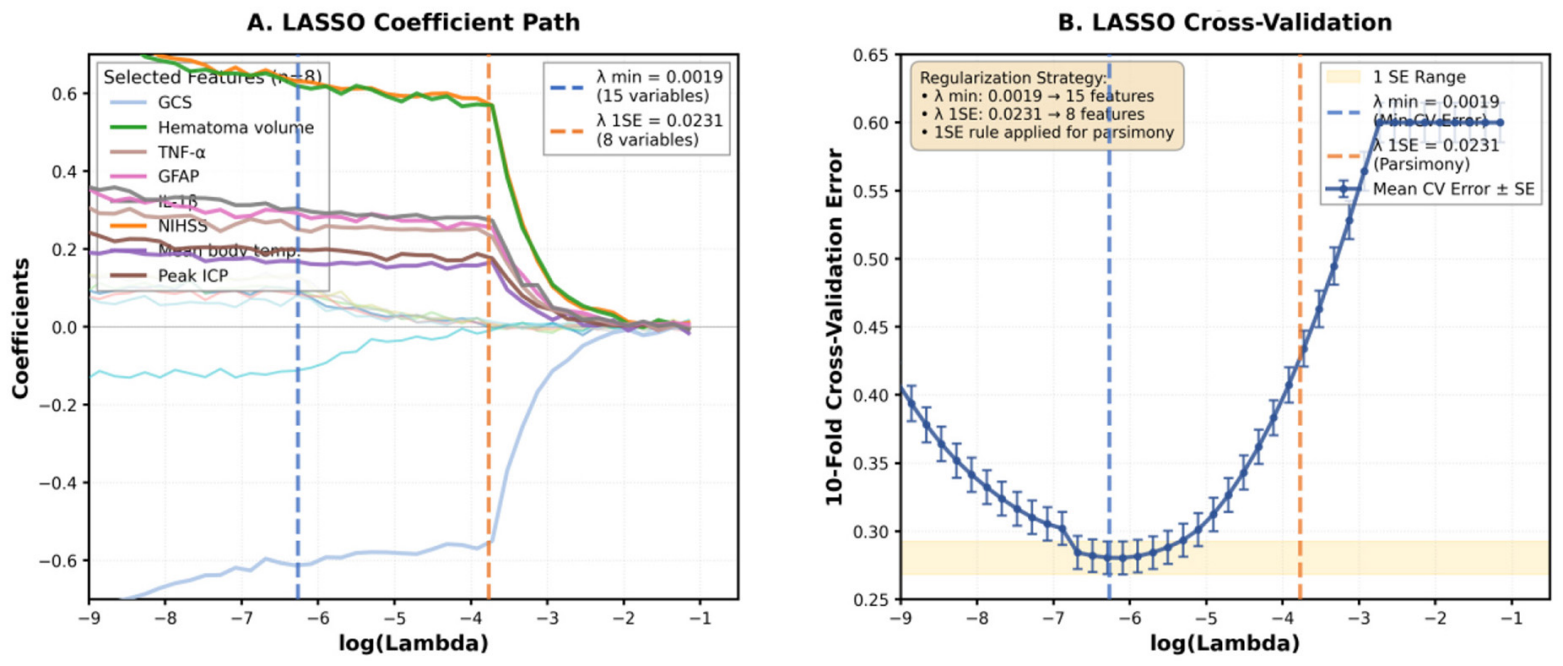
LASSO Feature Selection Analysis. **(A)** LASSO coefficient profiles of the 15 candidate variables. The y-axis represents the coefficient value, and the x-axis represents log (Lambda). **(B)** LASSO cross-validation curve showing the relationship between log (Lambda) and 10-fold cross-validation error. The blue dashed line indicates λmin = 0.0019 (15 variables), and the red dashed line indicates λ1SE = 0.0231 (8 variables selected). The yellow shaded area represents the one-standard-error range.

Using LASSO regression with 10-fold cross-validation on the training set, we systematically identified optimal predictive features from 15 candidate variables. Figure 1A illustrates the LASSO coefficient paths, showing how feature coefficients vary with the regularization parameter lambda (λ). As λ increases (moving right on the x-axis), coefficients shrink toward zero. Figure 1B presents the cross-validation error analysis. The model achieved minimal cross-validation error at λmin = 0.0019 (indicated by the blue dashed line), where all 15 variables were retained. Applying the one-standard-error rule for enhanced model parsimony, we selected the optimal λ1SE = 0.0231 (red dashed line). This threshold yielded a sparse model with 8 non-zero coefficients, maintaining predictive accuracy within one standard error of the minimum. The 8 features selected by LASSO at λ1SE included: admission GCS score, hematoma volume, TNF-α, GFAP, IL-1β, admission NIHSS score, mean body temperature, and peak intracranial pressure (ICP).

### 3.4 Machine learning model performance comparison

Five machine learning algorithms were trained on the training set (*n* = 199) using 10-fold cross-validation for hyperparameter optimization, then evaluated on the independent test set (*n* = 86). Table 2 summarizes the comprehensive performance metrics of all models on the test set.

**Table 2:** Performance comparison of machine learning models on the test set.

**Model**	**AUC (95% CI)**	**Accuracy**	**Sensitivity**	**Specificity**	**F1-score**	**PPV**	**NPV**
Random forest	0.883 (0.829–0.937)	0.824	0.829	0.818	0.836	0.843	0.818
Neural network	0.867 (0.809–0.925)	0.800	0.780	0.818	0.799	0.821	0.783
Decision tree	0.768 (0.692–0.844)	0.718	0.732	0.705	0.732	0.750	0.705
K-Nearest neighbors	0.745 (0.665–0.825)	0.694	0.683	0.705	0.705	0.726	0.689
Naïve bayes	0.731 (0.649–0.813)	0.682	0.659	0.705	0.693	0.711	0.667

The Random Forest (RF) model demonstrated superior performance with the highest AUC of 0.883 (95% CI: 0.829–0.937), significantly outperforming the Decision Tree (AUC = 0.768, DeLong test *P* = 0.003), K-Nearest Neighbors (AUC = 0.745, *P* = 0.001), and Naïve Bayes (AUC = 0.731, *P* = 0.001) models. The RF model also achieved the highest accuracy (82.4%), sensitivity (82.9%), F1-score (0.836), positive predictive value (84.3%), and negative predictive value (81.8%). The Neural Network model ranked second with an AUC of 0.867 (95% CI: 0.809–0.925), showing no significant difference from the RF model (DeLong test *P* = 0.312), but with slightly lower performance across all other metrics.

**Figure 2:**
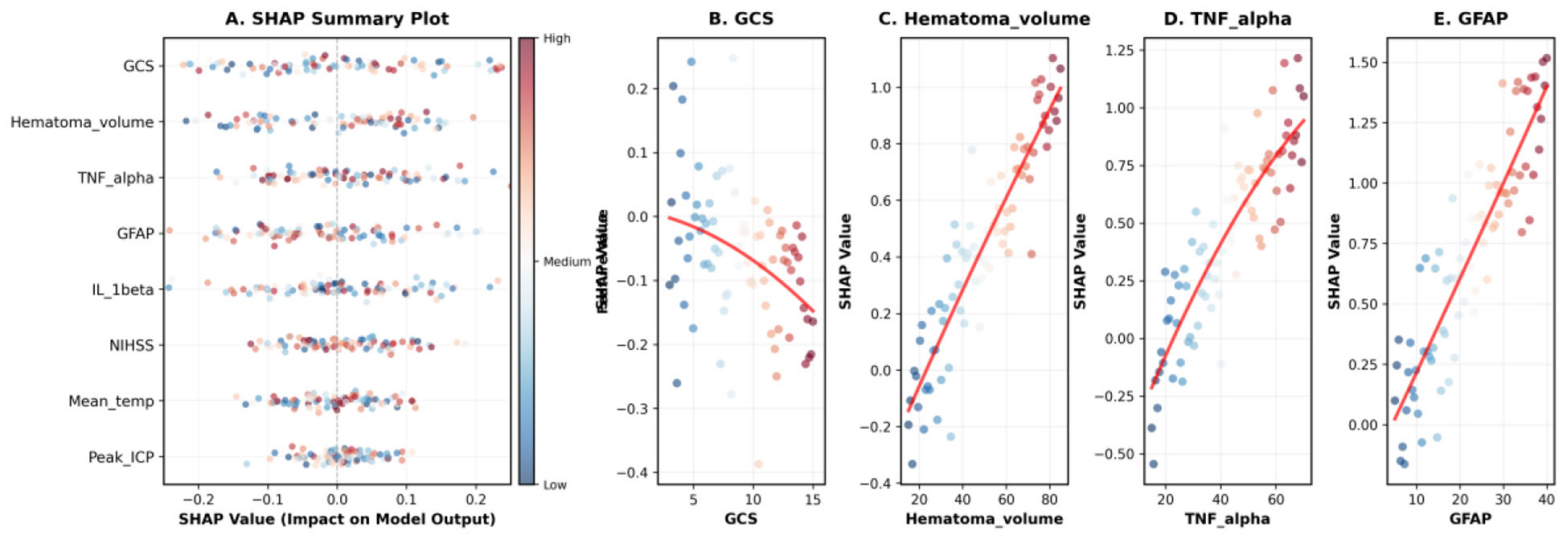
SHAP Analysis for Model Interpretability Based on LASSO-Selected Features. **(A)** SHAP summary plot showing the eight features selected by LASSO, ranked by mean absolute SHAP value.Each point represents a single patient, with color indicating the feature value (red = high, blue = low) and position along the x-axis indicating the SHAP value (impact on model prediction toward unfavorable outcome). The vertical gray dashed line at zero indicates no impact. **(B-E)** SHAP dependence plots for the four most important features: **(B)** admission GCS score showing strong inverse relationship with risk, **(C)** hematoma volume demonstrating positive linear relationship, **(D)** TNF-α exhibiting exponential risk increase above 40 pg/ml, and **(E)** GFAP showing threshold effect above 15 ng/ml. Red curves represent quadratic polynomial fits to visualize the non-linear relationships.

To enhance model interpretability and understand individual feature contributions, we employed SHapley Additive exPlanations (SHAP) analysis for the optimal Random Forest model. Figure 2A presents the SHAP summary plot for the eight LASSO-selected features, illustrating both the magnitude of importance and the directionality of impact. The vertical axis ranks features by their mean absolute SHAP value, while the horizontal axis shows the SHAP value (impact on model output). Each point represents a patient, with color indicating the feature value (red = high, blue = low).

The eight LASSO-selected features showed the following importance ranking: admission GCS score (mean |SHAP value| = 0.156), hematoma volume (0.142), TNF-α (0.128), GFAP (0.119), IL-1β (0.107), admission NIHSS score (0.098), mean body temperature (0.091), and peak intracranial pressure (0.085).

The SHAP dependence plots (Figures 2B–E) revealed the complex non-linear relationships between key features and outcome predictions. For admission GCS score (Figure 2B), there was a clear inverse relationship with risk, with particularly steep risk increases for GCS ≤ 8. Hematoma volume (Figure 2C) showed a near-linear positive relationship with SHAP values, with volumes >40 ml substantially increasing unfavorable outcome risk. TNF-α levels (Figure 2D) demonstrated an exponential relationship, with levels above 40 pg/ml associated with disproportionately higher risk. GFAP (Figure 2E) exhibited similar patterns, with a critical threshold around 15 ng/ml above which risk accelerated markedly.

ROC curves and calibration curves for four machine learning models in both training and validation sets. (A) ROC curves for the training set showing AUC values of 0.883 (Random Forest), 0.867 (Neural Network), 0.768 (Decision Tree), and 0.751 (Logistic Regression). (B) Calibration curves for the training set with 95% confidence intervals, demonstrating good agreement between predicted and observed probabilities. (C) ROC curves for the validation set with AUC values of 0.838 (Random Forest), 0.821 (Neural Network), 0.745 (Decision Tree), and 0.738 (Logistic Regression). (D) Calibration curves for the validation set, showing maintained calibration performance. The shaded green areas in calibration plots represent the acceptable ±5% deviation range from perfect calibration.

**Figure 3:**
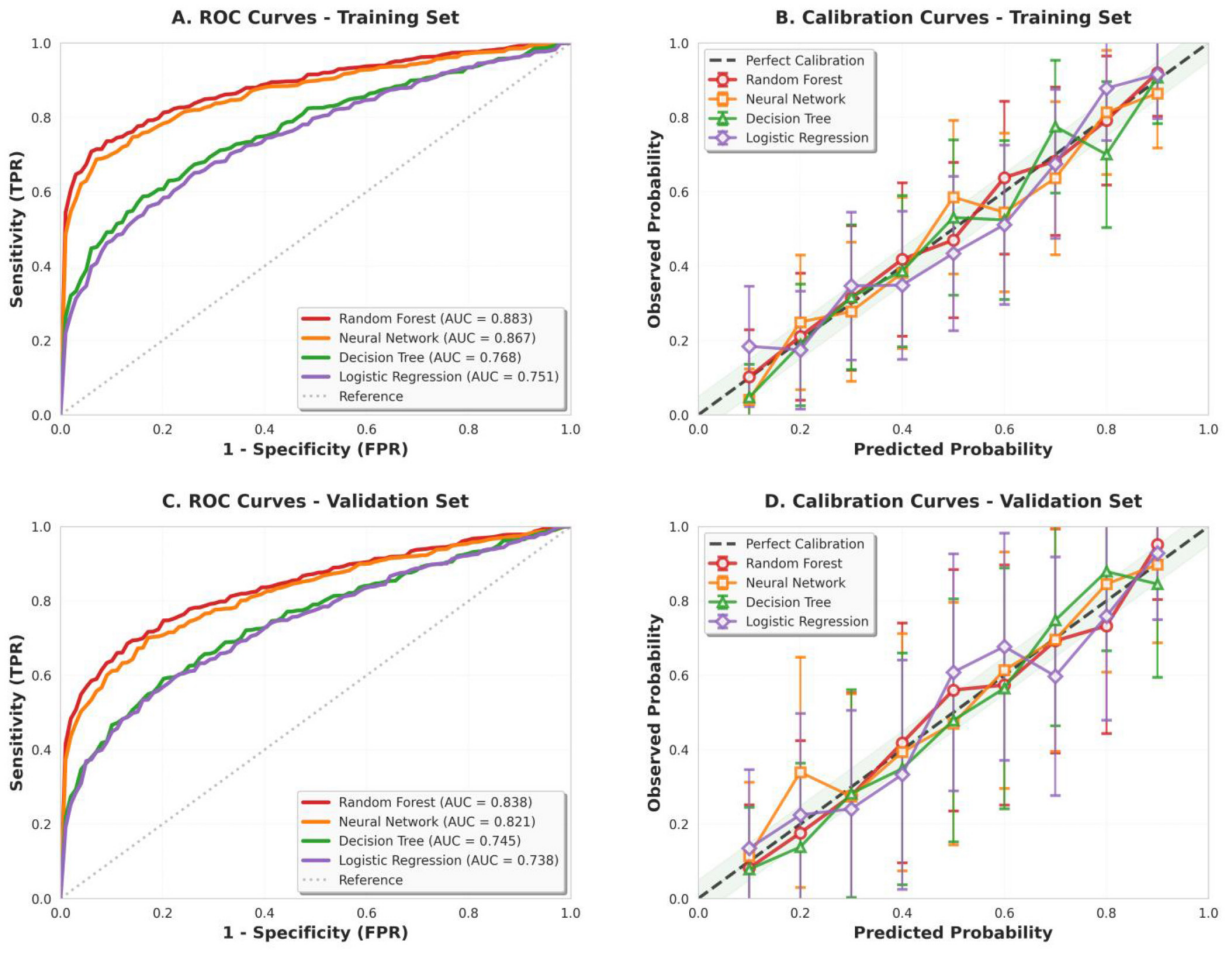
Comprehensive model performance comparison across training and validation sets.

To comprehensively evaluate model performance across both training and validation sets, we present ROC curves and calibration curves for all four machine learning models (Figure 3). The ROC curves (panels A and C) demonstrate the discriminative ability of each model, with Random Forest achieving the highest AUC values in both training (0.883) and validation (0.838) sets, followed by Neural Network (0.867 and 0.821, respectively). The calibration curves (panels B and D) assess the agreement between predicted probabilities and observed outcomes, with all models showing reasonable calibration within the acceptable ±5% range, particularly in the mid-probability range (0.3–0.7).

## Discussion

In this prospective, multicenter cohort of 285 surgically treated patients with spontaneous supratentorial deep intracerebral hemorrhage (sICH), we developed and validated multimodal machine-learning models for predicting functional outcome. The main findings are as follows: (1) patients with unfavorable outcomes (mRS 4–6) were older and presented with more severe neurological deficits (lower GCS and higher NIHSS), larger hematoma volumes, and higher rates of IVH and significant midline shift; (2) inflammatory markers (TNF-α, IL-1β, and NLR) and neuronal injury biomarkers (GFAP, S100β, and NSE) were significantly elevated in the unfavorable group; (3) LASSO feature selection identified an 8-variable parsimonious set (admission GCS, hematoma volume, TNF-α, GFAP, IL-1β, admission NIHSS, mean body temperature, and peak ICP); and (4) among five algorithms, the Random Forest model achieved the best overall performance in the independent test set (AUC 0.883, accuracy 0.824), and SHAP analysis provided clinically intuitive explanations, highlighting both directionality and potential non-linear threshold effects.

### Clinical and imaging determinants: consistency with established prognostic frameworks

The prominent roles of admission GCS, NIHSS, hematoma volume, IVH, and midline shift observed in our cohort are consistent with widely used clinical prognostic frameworks for ICH, which emphasize neurological severity and hemorrhage burden as principal drivers of outcome. In our data, unfavorable outcomes were associated with a markedly lower median GCS (8 vs. 11) and larger hematoma volume (45.8 vs. 32.6 ml), accompanied by more frequent IVH (≈68.5% vs. 41.9%) and midline shift >5 mm (≈74.6% vs. 51.6%). These findings reinforce the concept that early neurological status and mass effect reflect irreversible primary injury and the extent of secondary pathophysiological cascades, thereby shaping functional recovery potential even in surgically managed patients. Importantly, our cohort was restricted to surgically treated deep sICH, a subgroup for whom outcome prediction can be particularly challenging because perioperative management, timing of intervention, and intensive care may modify early trajectories. The maintenance of strong signal from these core clinical and imaging variables suggests that, even under aggressive treatment, baseline severity remains a dominant determinant of outcome (1, 2).

### Added value of inflammatory and neuronal injury biomarkers: capturing secondary injury biology

A major contribution of this study is the integration of systemic inflammatory markers and neuronal injury biomarkers with conventional clinical/imaging features. We found that TNF-α and IL-1β were substantially higher in patients with unfavorable outcomes, as were GFAP, S100β, and NSE, indicating that both inflammatory activation and brain tissue injury are closely linked with functional prognosis after sICH surgery. From a mechanistic perspective, ICH triggers rapid microglial activation and cytokine release, promoting blood–brain barrier disruption, perihematomal edema, and excitotoxicity (10, 11); concurrently, structural injury to astroglia and neurons releases GFAP, S100β, and NSE into the circulation. Our results support the hypothesis that circulating biomarkers can serve as accessible surrogates of these biological processes and provide incremental prognostic information beyond anatomy and bedside scores (12).

Notably, SHAP dependence patterns suggested non-linear relationships and potential “risk acceleration zones” for key biomarkers (e.g., TNF-α above ~40 pg/ml and GFAP above ~15 ng/ml), while hematoma volume demonstrated a near-linear increase in risk. These findings are clinically relevant because they imply that biomarker-informed thresholds may help identify patients at disproportionate risk of poor functional recovery (13, 14). However, because such thresholds are model- and cohort-dependent, they should be considered hypothesis-generating and require confirmation in external datasets, particularly given inter-assay variability and differences in sampling time windows.

### Physiological variables and modifiability: temperature and ICP as clinically actionable domains

The inclusion of mean body temperature and peak ICP among the LASSO-selected features highlights the importance of physiological derangements in the perioperative period. Hyperthermia can amplify metabolic demand and inflammatory injury, whereas elevated ICP reflects critical intracranial compliance failure and compromised perfusion (15). The finding that these parameters contributed materially to prediction suggests that outcome after surgically treated deep sICH is not determined solely by the initial hemorrhage burden, but also by secondary physiological insults that may be amenable to targeted management. Although our study is predictive rather than causal, these results support future work testing whether biomarker- or risk-stratified protocols for temperature control and ICP management can improve outcomes (16, 17).

### Model performance and interpretability: Random Forest superiority and SHAP-based clinical transparency

Among the five tested algorithms, Random Forest provided the best discriminative performance in the independent test set (AUC 0.883) and achieved balanced sensitivity and specificity (~0.83 and ~0.82, respectively), with the highest F1-score (0.836). Neural networks performed comparably in AUC (0.867) but were modestly inferior across other metrics, while simpler methods (Decision Tree, KNN, Naïve Bayes) showed lower AUCs and accuracies. These results are consistent with the advantages of ensemble tree methods in clinical datasets: robustness to noise, ability to model non-linearities and interactions, and good performance with heterogeneous variable types. Importantly, the use of SHAP addressed a common barrier to clinical adoption of machine-learning models by explaining feature contributions at both cohort and individual levels (18). The SHAP summary and dependence plots aligned with clinical intuition (e.g., lower GCS and larger hematoma volume increasing risk) while revealing higher-order patterns (e.g., biomarker thresholds), improving transparency and potentially facilitating clinical communication and decision support (19, 20).

### Strengths, limitations, and future directions

This study has several strengths: a prospective, multicenter design; inclusion of a clinically important but less-studied subgroup (surgically treated deep sICH); multimodal feature integration spanning clinical, imaging, physiological monitoring, and biomarkers; and interpretable modeling using SHAP. Nevertheless, limitations should be acknowledged. First, the sample size, while reasonable for a prospective surgical cohort, may still limit the complexity of models and the stability of threshold effects; larger external validations are needed. Second, the cohort was restricted to surgically treated deep sICH, which may limit generalizability to conservatively treated patients or lobar/infratentorial hemorrhages. Third, biomarkers were measured at a single early time point; dynamic trajectories may better capture evolving secondary injury and could further improve prediction. Fourth, despite calibration curve assessments, future studies should incorporate decision-curve analysis to quantify clinical net benefit across threshold probabilities and evaluate the model's utility for specific decisions (e.g., intensive monitoring, resource allocation, or tailored rehabilitation planning).

## Conclusions

In surgically treated patients with supratentorial deep ICH, a machine-learning approach integrating clinical severity, hemorrhage burden, physiological parameters, and inflammatory/neuronal injury biomarkers achieved good discrimination for functional outcome prediction, with Random Forest outperforming alternative algorithms. SHAP-based interpretability highlighted admission GCS and hematoma volume as dominant predictors and underscored the prognostic relevance of inflammatory and neuronal injury biomarkers, suggesting that multimodal, explainable models may facilitate preoperative risk stratification and individualized perioperative management.

## Data Availability

The raw data supporting the conclusions of this article will be made available by the authors, without undue reservation.
